# The Research on the Treatment of Metastatic Skin Cutaneous Melanoma by Huanglian Jiedu Decoction Based on the Analysis of Immune Infiltration Analysis

**DOI:** 10.1155/2021/9952060

**Published:** 2021-06-21

**Authors:** Ding Li, Shi-Fang Li, Xiao-Yuan Li, Xiao-Wei Sun, Tian-Yue Sun, Guo-Jie Hu

**Affiliations:** ^1^Department of Traditional Chinese Medicine, The Affiliated Hospital of Qingdao University, Qingdao, Shandong 26600, China; ^2^Department of Neurosurgery, The Affiliated Hospital of Qingdao University, Qingdao, Shandong 26600, China; ^3^Basic Medical School, Medical College, Qingdao University, Qingdao, Shandong 26600, China

## Abstract

**Objective:**

To explore the potential mechanism of Huanglian Jiedu Decoction (HJD) treatment and prevention of metastatic Cutaneous Melanoma (CM) occurrence and metastasis based on network pharmacological methods and immune infiltration analysis.

**Methods:**

The GEO database was used to obtain metastatic CM disease targets, the TCMSP database and the HERB database were used to obtain HJD action targets, core genes were screened by protein interaction network, and the potential mechanism of HJD in the treatment of metastatic CM was explored by enrichment analysis, prognostic analysis and immune infiltration analysis.

**Results:**

HJD treatment of metastatic CM involved 60 targets, enrichment analysis showed that HJD treatment of metastatic CM involved Chemokine signaling pathway, NF-kappa B signaling pathway, and Fluid shear stress and atherosclerosis, etc. Prognostic analysis revealed that HJD had a certain ability to improve the prognosis of metastatic CM patients. Immune infiltration analysis showed that HJD could inhibit the immune cell infiltration of metastatic CM patients by acting on related targets.

**Conclusions:**

Our study identified the potential mechanism of HJD in the treatment of metastatic CM through network pharmacology, and revealed the mechanism of HJD in the prevention of Skin Cutaneous Melanoma metastasis through immune infiltration analysis and prognostic analysis.

## 1. Introduction

Cutaneous Melanoma (CM) is one of the most common, aggressive and dangerous cancer in cutaneous tissues [1]. According to the latest cancer statistics, 106110 cases are estimated to be diagnosed as CM in 2021, it is estimated that there will be 7180 deaths due to the CM in 2021 [2]. However, patients with CM usually metastasize because of the highly aggressive nature of CM. The prognosis of patients is poor when CM patients turn to an metastatic cutaneous melanoma patient [3]. When CM in the advanced stage of the disease or have metastasis, the prognosis is poor [4]. The median survival of patients with metastatic CM is less than 1 year [5]. Thus, these sobering data illustrate a critical need for novel therapy and alternative therapy of CM. An increasing number of research have found that Traditional Chinese Medicine (TCM) play an important role in alleviating patients' symptoms, controlling the growth of tumors, preventing metastasis of tumor and prolongs the survival of tumor patients [6].

Huanglian jiedu decoction (HJD), a well-known and classical Chinese herbal formula recorded in the Tang dynasty, is composed of Huanglian (Coptidis rhizoma), Huangqin (Scutellariae radix), Huangbai (Phellodendri Chinensis cortex) and Zhizi (Gardenia fructus). Increasing evidences reveal that HJD has anti-inflammatory, anti-allergic, anti-microbial and anti-tumor effects in clinical practice [7]. Due to the characteristics of a multi-drug and multi-component traditional Chinese medicine compound, we still do not know the main mechanism of HJD in treating cancer. According to a study on HJD clinical practice, the patients with cancer were shown to have reduced tumor growth and metastasis after HJD treatment [8]. Based on the data of main components of HJD from the TCMSP database, such as geniposide, quercetin, berberine and palmatine, could target on tumor-related genes spontaneously. HL's research shows that Baicalein and Baicalin derived from Huangqin inhibit the occurrence and growth of tumors in melanoma models via Metabolic Inhibition [9]. Related studies have also proved that Huangqin can inhibit the occurrence, development and metastasis of melanoma through the ERK signaling pathway and the P13K/AKT signaling pathway [10, 11]. It has been proven that Huanglian can accelerate the intrinsic apoptosis of melanoma cells by activating BAX and BAK [12]. By acting on p38, GR and DHODH to inhibit the proliferation of melanoma cells [13]. Inhibit the invasion and migration ability of melanoma cells by acting on the P13K/AKT signaling pathway [14]. Huangbai is also believed to work together with Huanglian in anti-inflammatory effects [15]. The study of Akihisa T and Im M found that Zhizi can inhibit the production of melanin in melanoma with almost no toxicity to the cells. And can inhibit the invasion ability of melanoma through NF-*κ*B and HIF-1*α* signal pathway [16, 17]. The anti-tumor effect of each drug has been reported in previous research and these compounds have been verified to inhibit the growth and metastasis of cancer. The diversified anti-cancer mechanisms thanks to the multi-component increase the possibility of these compounds intervention different signaling pathways involved in cancer metastatic.

The aim of this study is through bioinformatics to explore the potential mechanism of HJD to prevent the transfer of CM and to improve the poor prognosis of CM. Our study may reveal HJD-intervened molecular mechanisms in the tumorigenesis and metastasis of CM and provide additional choice for the novel therapy for CM.

## 2. Methods and Materials

### 2.1. Metastatic CM Differentially Expressed Gene Screening

The Gene Expression Omnibus database (GEO, https://www.ncbi.nlm.nih.gov/geo) is an international public functional genomics data repository contained high throughout gene expression data., chips and microarrays [18]. The four gene expression datasets of metastatic CM (GSE7553 [19], GSE8401 [20], GSE15605 [21], GSE46517 [22]) were obtained by the GEO database contained 177 metastasis skin cutaneous melanoma tissue samples and 122 primary skin cutaneous melanoma tissue samples (Table 1). We performed the differential analysis (|Log2FC| > 1, adjusted *p* value < 0.05) by comparing metastatic CM tissues and CM tissues in the *R* computing environment using limma package to obtain differential expressed genes (DEGs).

### 2.2. HJD Related Target Screening

HERB database (https://herb.ac.cn/) is a high-throughput experiment and reference-guided database of TCM. This database provides 6164 gene expression profiles from 1037 high-throughput experiments evaluating TCM herbs and ingredients [23]. In the herb database, we search related gene targets of each drug of HJD and collect them.

Traditional Chinese Medicine Systems Pharmacology Database and Analysis Platform (TCMSP, https://tcmspw.com/tcmsp.php) is a unique systems pharmacology platform of TCM that contains the relationships between drugs, targets and diseases. The TCMSP database offers 29,384 ingredients, 3,311 targets and 837 associated diseases [24]. In TCMSP databases, we search related ingredients and related gene targets of each drug of HJD, cut value were set as oral bioavailability (OB) ≥ 30% and drug-likeness (DL) ≥ 0.18.

### 2.3. Obtain the Intersection of HJD Target Genes and DEGs

We use “VENN” package (version 1.9, https://mirrors.tuna.tsinghua.edu.cn/CRAN/web/packages/venn/index.html) in *R* studio software to extract the intersection between DEGs and related gene targets of HJD, and the results was visualized. The target genes of intersection was considered to be the target genes involved in HJD treatment of metastatic CM.

### 2.4. Construction of PPI Network and Screening of Core Genes

STRING database (https://string-db.org/) offers protein-protein association data for a large number of organisms [25]. In the STRING database, we search the data of protein-protein interaction of DEGs, and construct the protein-protein interaction network (PPI), organism was set as “*Homo sapiens*.” We used “CytoHubba” package (version 0.1, https://apps.cytoscape.org/apps/cytohubba) in Cytoscape software (version 3.7.2, https://cytoscape.org/) to determine the core genes in PPI, “MCC” was set as the calculation method. In order to understand the correlation between the expression of 10 core genes. We collect the expression of core genes in each patient and obtain potentially relevant information about core genes in metastatic CM patients via correlation analysis.

### 2.5. GO and KEGG Enrichment Analysis

We performed enrichment analysis on core genes by “clusterProfiler” package (version 3.18.0, https://bioconductor.org/packages/release/bioc/html/clusterProfiler.html) in *R* studio software, to get the biological processes and signal pathways involved in obtaining HJD to treat metastatic CM.

### 2.6. Immune Infiltration Analysis

Tumor IMmune Estimation Resource database (TIMER, https://cistrome.shinyapps.io/timer/) is a comprehensive analysis of tumor-infiltrating immune cells tool that could offer various analyses with the dataset of 10897 samples [26]. In this study, We analyzed the expression and prognosis of 10 core genes in metastatic CM through the TIMER database and the correlation between the expression of 10 core genes and the abundance of immune cells and tumor purity. In order to obtain the immune mechanism that may be involved in HJD intervention and treatment of metastatic CM. 10 core genes expression and its correlation with the abundance of immune cells were evaluated using spearman's correlation, and cancer type was set as SKCM-metastasis. The infiltration level for each somatic copy number alterations (SCNA) category was compared with the normal using a two-sided Wilcoxon rank-sum test.

### 2.7. Prognostic Analysis

We performed the prognostic analysis to explore the potential relationship between the metastatic CM patient prognosis and indicators (the expression of core genes and the abundance of 6 immune cells) to provide evidence that HJD may improve the prognosis of metastatic CM patients. The cancer type was set as SKCM-metastasis.

## 3. Results

### 3.1. The Result of Difference Analysis

**Figure 1:**
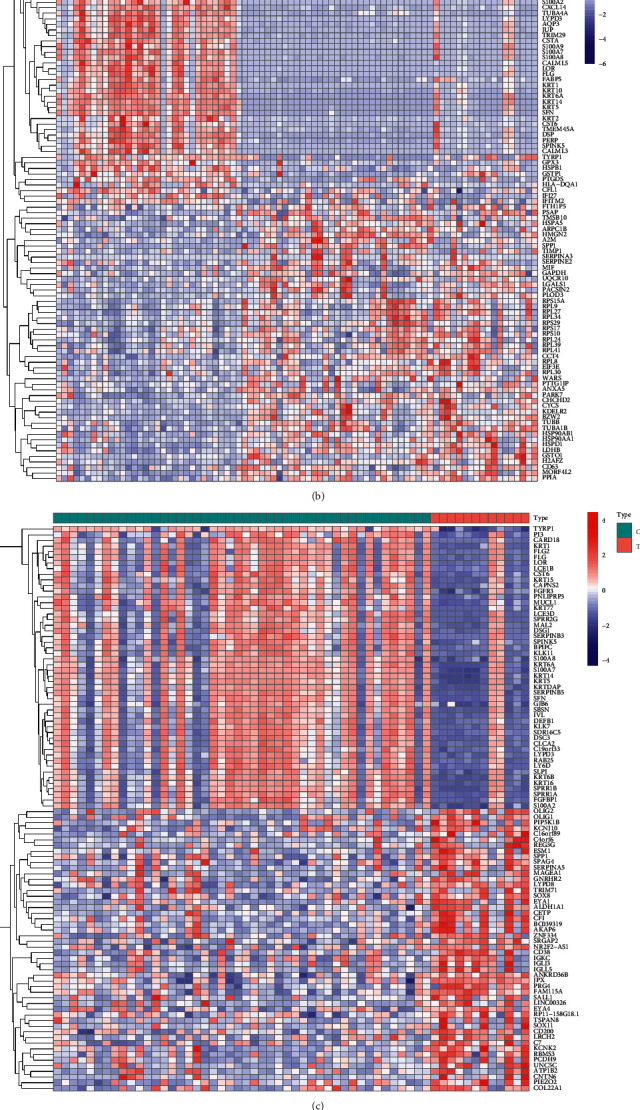
The result of differentially analysis is visualized by the heatmap. (a) Part (GSE7553), (b) part (GSE8401), (c) part (GSE15605), (d) part (GSE46517). The gradual color ranging from blue to red represents the changing process from down-to up-regulation.

**Figure 2:**
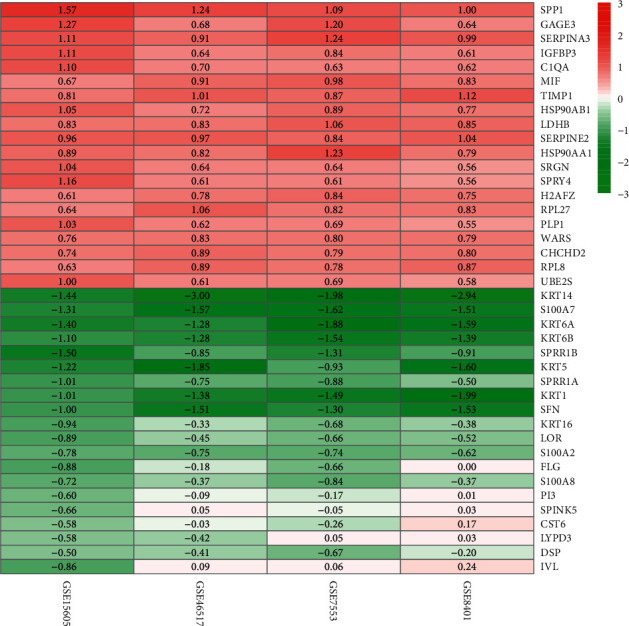
Merging and visualization based on the results of the 4 data sets difference analysis. The gradual color ranging from green to red represents the changing process from down-to up-regulation.

We performed differential analysis on four GEO datasets (Figure 1), and merge the results of the different analyses of the 4 data sets (Figure 2). A total of 1635 differentially expressed genes were screened by differentially analysis, including 896 down-regulated genes and 739 up-regulated genes.

### 3.2. The Result of HJD Target Genes

**Figure 3:**
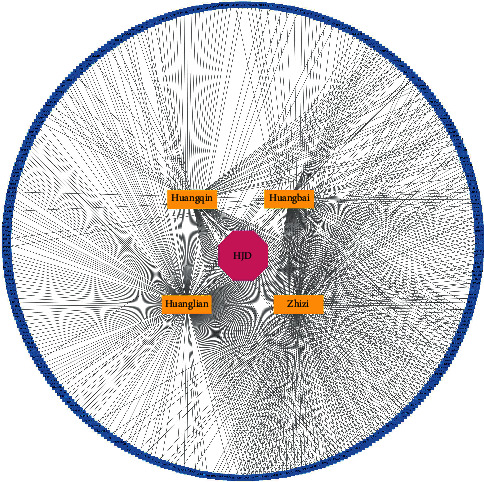
HJD-drug-target network including 1 Chinese herbal formula, 4 Chinese medicines and 376 targets.

We screened HJD target genes in two TCM databases (TCMSP database and HERB database). In the HERB database, a total of 283 HJD target genes was screened, and 176 HJD target genes were screen from the TCMSP database. A total of 376 HJD genes were obtained after removing the repetitive parts (Figure 3).

### 3.3. The Result of the Intersection of HJD and DEGs Showed by Venn Diagram

**Figure 4:**
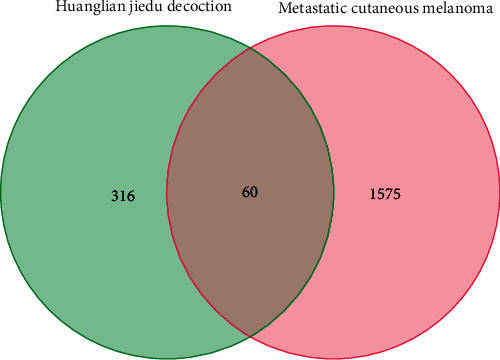
The intersection between HJD and DEGs. A total of 60 genes have been screened out.

we used “VENN” package in *R* studio software to extract the intersection between HJD and DEGs, and a total of 60 genes were screened (Figure 4).

### 3.4. The Result of the Construction of the PPI Interaction Network and Screening of Core Genes

**Figure 5:**
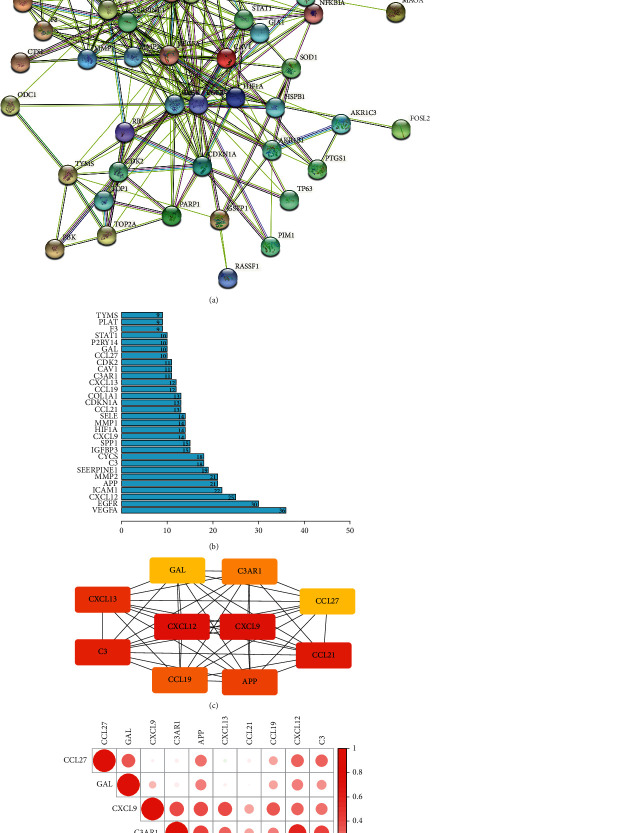
Construction the PPI network through STRING database (a). Rank based on the number of nodes in the PPI network (b). Top 10 core genes was screened out based on “MCC” calculation method (c). Correlation analysis between the expression of 10 core genes. The expression of C3 and C3AR1, the expression of CXCL13 and CCL19, the expression of CCL21 and CXCL12, and the expression of CCL19 and C3 were significantly positively correlated (d).

Based on the intersection between HJD and DEGs, we constructed the PPI network through the STRING database (Figure 5(a)), and screen out the core gene (CXCL12, CXCL9, CXCL13, CCL21, CCL27, CCL19, APP, C3, GAL, C3AR1) of HJD intervention in metastatic CM by Cytoscape software (Figure 5(c), Table 2). According to the result of correlation analysis, we found that the potentially relevant information between 10 core genes. The expression of C3 and C3AR1, the expression of CXCL13 and CCL19, the expression of CCL21 and CXCL12, and the expression of CCL19 and C3 were significantly positively correlated (Figure 5(d)).

### 3.5. The Result of Enrichment Analysis

**Figure 6:**
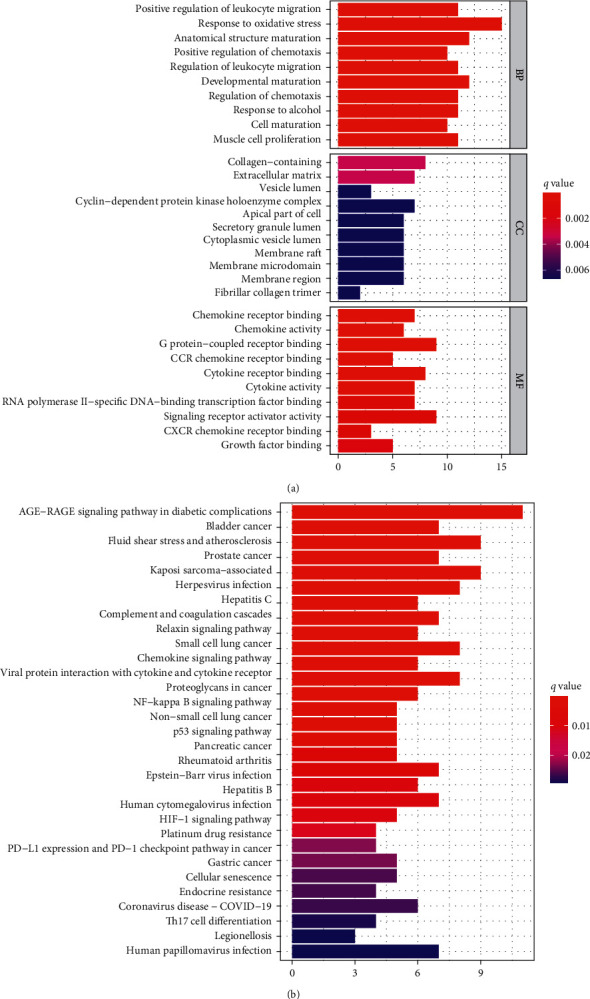
Enrichment analysis of HJD treat METASTATIC CM. Cellular components, biological processes, and molecular functions analysis (a). KEGG pathway analysis (b).

We obtained the potential mechanism of HJD treatment intervention metastatic CM through enrichment analysis (GO enrichment and KEGG enrichment) of the intersection between HJD and metastatic CM. Biological process (BP) analysis suggested that HJD treat metastatic CM were associated with positive regulation of leukocyte migration, response to oxidative stress, anatomical structure maturation (Figure 6(a)). Cellular component (CC) analysis suggested that HJD treat metastatic CM were involved in the collagen-containing extracellular matrix, vesicle lumen, and apical part of the cell (Figure 6(a)). Moreover, molecular function (MF) analysis revealed that HJD treat metastatic CM were enriched in chemokine receptor binding, G protein-coupled receptor binding, and cytokine receptor binding (Figure 6(a)). Result of Kyoto Encyclopedia of Genes and Genomes (KEGG) revealed that HJD treat metastatic CM were enriched in Chemokine signaling pathway, NF-kappa B signaling pathway, and Fluid shear stress and atherosclerosis, etc. (Figure 6(b)).

### 3.6. The Results of the Correlation between the Expression of Core Genes in Metastatic CM and the Level of Immune Infiltration

**Figure 7:**
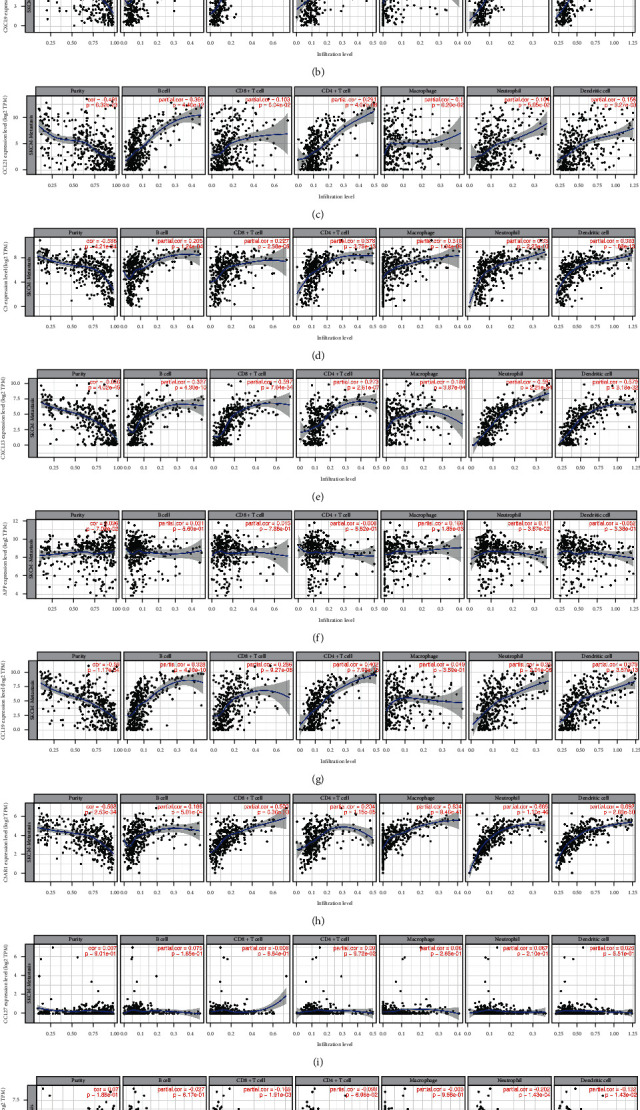
Correlation of core genes expression with immune infiltration level in METASTATIC CM (TIMER). (a) The expression of CXCL12 is positively correlated with the level of B cell (partial.cor = 0.136, *p*=1.15*e* − 02), CD8+ T cell (partial.cor = 0.344, *p*=9.29*e* − 11), CD4+ T cell (partial.cor = 0.28, *p*=1.24*e* − 07), macrophage (partial.cor = 0.467, *p*=2.16*e* − 20), neutrophil (partial.cor = 0.408, *p*=1.62*e* − 15) and dendritic cell (partial.cor = 0.444, *p*=4.92*e* − 18) immune infiltration. Negatively correlated with the level of immune infiltration of purity (cor = -0.615, *p*=2.59*e* − 38). (b) The expression of CXCL9 is positively correlated with the level of B cell (partial.cor = 0.201, *p*=1.73*e* − 04), CD8+ T cell (partial.cor = 0.679, *p*=8.20*e* − 47), CD4+ T cell (partial.cor = 0.255, *p*=1.71*e* − 06), macrophage (partial.cor = 0.255, *p*=1.27*e* − 06), neutrophil (partial.cor = 0.68, *p*=6.05*e* − 49) and dendritic cell (partial.cor = 0.672, *p*=1.48*e* − 46) immune infiltration. Negatively correlated with the level of immune infiltration of purity (cor = -0.585, *p*=5.42*e* − 34). (c) The expression of CCL21 is positively correlated with the level of B cell (partial.cor = 0.361, *p*=4.46*e* − 12), CD8+ T cell (partial.cor = 0.103, *p*=6.04*e* − 02), CD4+ T cell (partial.cor = 0.291, *p*=4.07*e* − 08), macrophage (partial.cor = 0.1, *p*=6.20*e* − 02), neutrophil (partial.cor = 0.104, *p*=5.05*e* − 02) and dendritic cell (partial.cor = 0.158, *p*=3.27*e* − 03) immune infiltration. Negatively correlated with the level of immune infiltration of purity (cor = -0.491, *p*=6.32*e* − 23). (d) The expression of C3 is positively correlated with the level of B cell (partial.cor = 0.205, *p*=1.24*e* − 04), CD8+ T cell (partial.cor = 0.227, *p*=2.58*e* − 05), CD4+ T cell (partial.cor = 0.378, *p*=3.75*e* − 13), macrophage (partial.cor = 0.318, *p*=1.04*e* − 09), neutrophil (partial.cor = 0.33, *p*=2.23*e* − 10) and dendritic cell (partial.cor = 0.383, *p*=1.88*e* − 13) immune infiltration. Negatively correlated with the level of immune infiltration of purity (cor = -0.586, *p*=4.21*e* − 34). (e) The expression of CXCL13 is positively correlated with the level of B cell (partial.cor = 0.327, *p*=4.30*e* − 10), CD8+ T cell (partial.cor = 0.597, *p*=7.04*e* − 34), CD4+ T cell (partial.cor = 0.273, *p*=2.61*e* − 07), macrophage (partial.cor = 0.188, *p*=3.87*e* − 04), neutrophil (partial.cor = 0.591, *p*=2.21*e* − 34) and dendritic cell (partial.cor = 0.579, *p*=3.18*e* − 32) immune infiltration. Negatively correlated with the level of immune infiltration of purity (cor = -0.656, *p*=4.02*e* − 45). (f) The expression of APP is positively correlated with the level of B cell (partial.cor = 0.031, *p*=5.60*e* − 01), CD8+ T cell (partial.cor = 0.015, *p*=7.88*e* − 01), macrophage (partial.cor = 0.166, *p*=1.85*e* − 03), neutrophil (partial.cor = 0.11, *p*=3.87*e* − 02)and purity (cor = 0.096, *p*=7.08*e* − 02) immune infiltration. Negatively correlated with the level of immune infiltration of CD4+ T cell (partial.cor = -0.008, *p*=8.82*e* − 01) and dendritic cell (cor = -0.052, *p*=3.38*e* − 01). (g) The expression of CCL19 is positively correlated with the level of B cell (partial.cor = 0.328, *p*=4.10*e* − 10), CD8+ T cell (partial.cor = 0.286, *p*=9.27*e* − 08), CD4+ T cell (partial.cor = 0.402, *p*=7.93*e* − 15), macrophage (partial.cor = 0.049, *p*=3.59*e* − 01), neutrophil (partial.cor = 0.25, *p*=2.01*e* − 06) and dendritic cell (partial.cor = 0.379, *p*=3.57*e* − 13) immune infiltration. Negatively correlated with the level of immune infiltration of purity (cor = -0.59, *p*=1.17*e* − 34). (h) The expression of C3AR1 is positively correlated with the level of B cell (partial.cor = 0.186, *p*=5.01*e* − 04), CD8+ T cell (partial.cor = 0.503, *p*=6.36*e* − 23), CD4+ T cell (partial.cor = 0.234, *p*=1.15*e* − 05), macrophage (partial.cor = 0.634, *p*=8.46*e* − 41), neutrophil (partial.cor = 0.668, *p*=1.10*e* − 46) and dendritic cell (partial.cor = 0.692, *p*=2.69*e* − 50) immune infiltration. Negatively correlated with the level of immune infiltration of purity (cor = -0.588, *p*=2.53*e* − 34). (i) The expression of CCL27 is positively correlated with the level of B cell (partial.cor = 0.075, *p*=1.65*e* − 01), CD4+ T cell (partial.cor = 0.09, *p*=9.72*e* − 02), macrophage (partial.cor = 0.06, *p*=2.66*e* − 01), neutrophil (partial.cor = 0.0.067 *p*=2.10*e* − 01), dendritic cell (partial.cor = 0.025, *p*=6.51*e* − 01) and purity (cor = 0.007, *p*=9.01*e* − 01) immune infiltration. Negatively correlated with the level of immune infiltration of CD8+ T cell (partial.cor = -0.008, *p*=8.84*e* − 01). (j) The expression of GAL is positively correlated with the level of purity (cor = 0.07, *p*=1.88*e* − 01) immune infiltration. Negatively correlated with the level of immune infiltration of B cell (partial.cor = -0.027, *p*=6.17*e* − 01), CD8+ T cell (partial.cor = -0.169, *p*=1.91*e* − 03), CD4+ T cell (partial.cor = -0.098, *p*=6.96*e* − 02), macrophage (partial.cor = -0.003, *p*=9.58*e* − 01), neutrophil (partial.cor = -0.202, *p*=1.43*e* − 04) and dendritic cell (partial.cor = -0.132, *p*=1.43*e* − 02).

**Figure 8:**
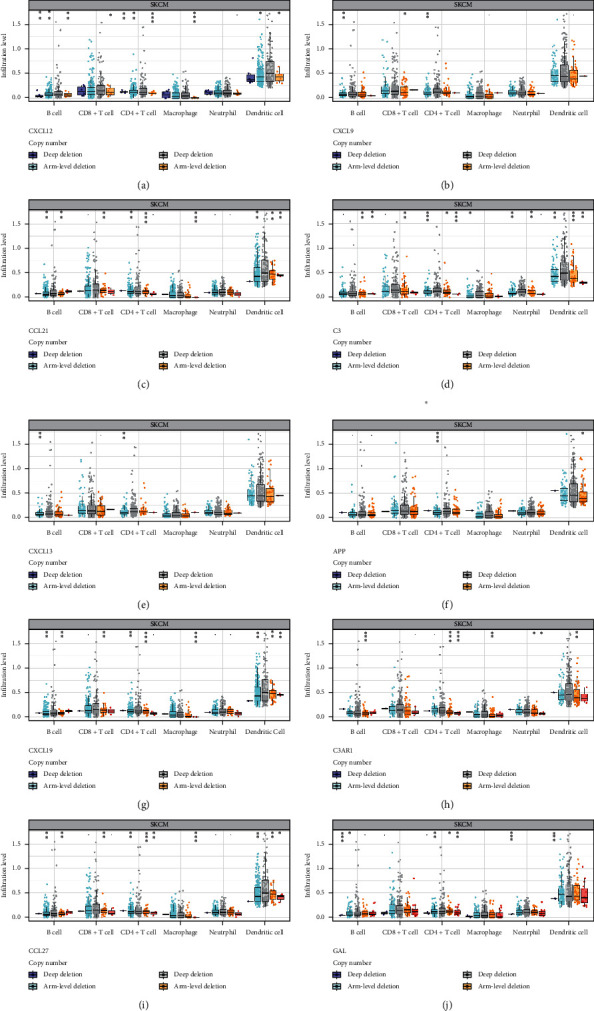
(a) The gene CXCL12 existed deep deletion, arm-level deletion, diplod and arm-level gain in METASTATIC CM. (b) The gene CXCL9 existed arm-level deletion, diploid, arm-level gain and high amplication. (c) The gene CCL21 existed deep deletion, arm-level deletion, diplod, arm-level gain and high amplication in METASTATIC CM. (d) The gene C3 existed arm-level deletion, diplod, arm-level gain and high amplication in METASTATIC CM. (e) The gene CXCL13 existed arm-level deletion, diplod, arm-level gain and high amplication in METASTATIC CM. (f) The gene APP existed deep deletion, arm-level deletion, diplod and arm-level gain in METASTATIC CM. (g) The gene CCL19 existed deep deletion, arm-level deletion, diplod, arm-level gain and high amplication in METASTATIC CM. (h) The gene C3AR1 existed deep deletion, arm-level deletion, diplod, arm-level gain and high amplication in METASTATIC CM. (i) The gene CCL27 existed deep deletion, arm-level deletion, diplod, arm-level gain and high amplication in METASTATIC CM. (j) The gene GAL existed deep deletion, arm-level deletion, diplod, arm-level gain and high amplication in METASTATIC CM.

As shown in Figure 7, GAL showed significant correlation with the abundance of CD8+ cell, CD4+ cell, neutrophil, and dendritic cell (Figure 7(j)). As for APP, significant correlations were obtained between gene expression and the abundance of macrophage and neutrophil (Figure 7(f)). The expression of CXCL12, CXCL9, CCL21, C3, CXCL13, C3AR1 was associated with the abundance of these six immune cells (B cell, CD8+ cell, CD4+ cell, macrophage, neutrophil, and dendritic cell) (Figures 7(a)–7(e), 7(h)). But CCL27 has nothing to do with the abundance of these six immune cells (Figure 7(i)). Except for macrophage, CCL19 was positively correlated with the abundance of the other immune cells (B cell, CD8+ cell, CD4+ cell, neutrophil, and dendritic cell) (Figure 7(g)). Moreover, somatic copy number alterations of core genes could certainly inhibit the immune cell infiltrations in metastatic CM (Figure 8).

### 3.7. The Result of Survival Analysis

**Figure 9:**
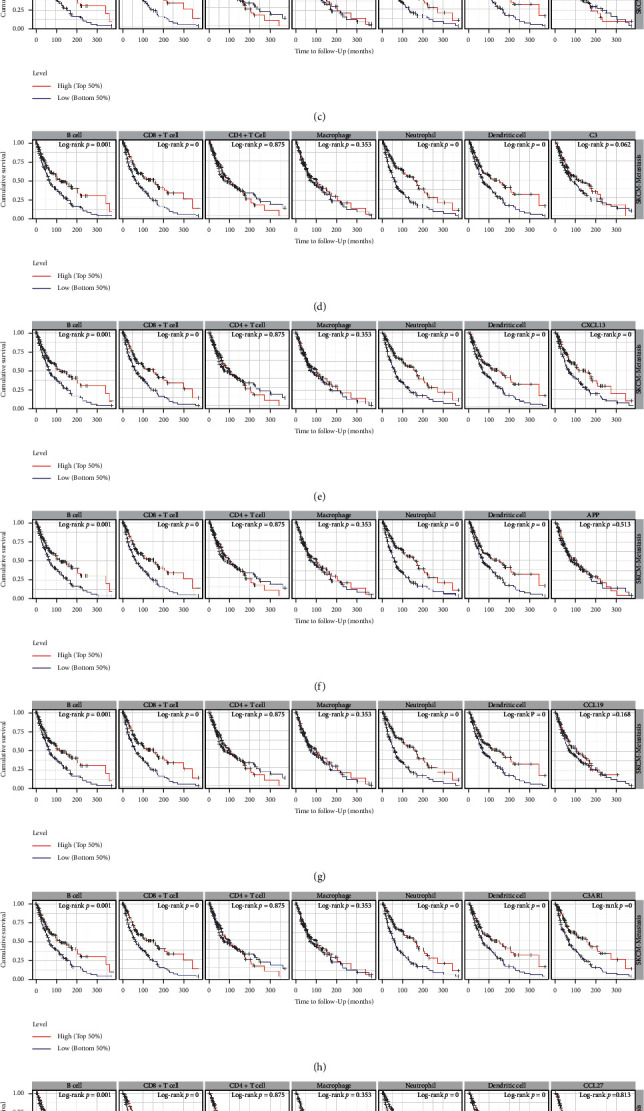
The prognostic value of core genes and immune cells in METASTATIC CM (TIMER). (a) METASTATIC CM patients with low expression of CXCL12 have a poor prognosis (*p* value = 0.12). (b) METASTATIC CM patients with low expression of CXCL9 have a poor prognosis (*p* value = 0). (c) METASTATIC CM patients with low expression of CCL21 have a poor prognosis (*p* value = 0.807). (d) METASTATIC CM patients with high expression of C3 have a poor prognosis (*p* value = 0.062). (e) METASTATIC CM patients with low expression of CXCL13 have a poor prognosis (*p* value = 0). (f) METASTATIC CM patients with high expression of APP have a poor prognosis (*p* value = 0.513). (g) METASTATIC CM patients with low expression of CCL19 have a poor prognosis (*p* value = 0.168). (h) METASTATIC CM patients with low expression of C3AR1 have a poor prognosis (*p* value = 0). (i) METASTATIC CM patients with high expression of CCL27 have a poor prognosis (*p* value = 0.813). (j) METASTATIC CM patients with low expression of GAL have a poor prognosis (*p* value = 0.001). Low-abundance B cells (*p* value = 0.001), CD8 + cells (*p* value = 0), macrophage (*p* value = 0.353), neutrophils (*p* value = 0) and dendritic cells (*p* value = 0) and high-abundance CD4+T cell (*p* value = 0.875) have a poor prognosis.

We then evaluated the association between core genes and the prognosis of METASTATIC CM patients. And the result showed that cumulative survival of metastatic CM patients with high level C3AR1, CXCL13, CXCL9, B cells, CD8+ cells, neutrophil, and dendritic cells were better compared with low/medium level (Figures 9(b), 9(d), 9(g)). And cumulative survival of metastatic CM patients with low level GAL was better compared with high/medium level (Figure 9(i)). The other core genes and immune cells would not affect the cumulative survival of METASTATIC CM patients.

## 4. Discussion

Metastatic Cutaneous Melanoma originating from melanocytes is one of the deadliest malignant tumor, historically carries a grim prognosis, with a median survival of 9 months and a long-term survival rate of 10% [27]. The tumorigenesis and metastatic of skin cutaneous melanoma is a multilevel, multistep, complex process associated with an interaction of exogenous and endogenous events and polygenic variation [28]. Thus, early detection and reasonable therapy are essential for patients who may have CM metastasis. These sobering data illustrate a critical need for novel and reasonable related to the therapy of metastatic CM. And our study is performed.

In recent years, with the in-depth study of Chinese medicine, an increasing number have found TCM plays an unexpected role in the prevention and treatment of tumors. Due to the character of the multi-drug and multi-component characteristics of the TCM compound formula, HJD as one of the classic TCM compounds formula plays an active role in the prevention and treatment of cancer [29]. At present, related studies have shown that the main compound (baicalin [30], baicalein [31], berberine [32], wogonin [33]) of HJD have anti-tumor effects. The study of Zhou et al. [34] and Chan et al. [35] prove that HJD in the treatment of hepatocellular carcinoma and non-small cell lung cancer can improve the prognosis of patients, improve the quality of life of patients and reduce the side effects of other therapies for patients.

We firstly compare metastatic CM and CM through differential analysis to obtain DEGs that are differentially expressed in METASTATIC CM, and Obtain the target of HJD through the TCMSP database and HERB database. Finally, screen out the intersection between targets of HJD and DEGs, and obtain 10 core genes by the “MCC” calculation method. As a result, a total of 10 core genes was obtained (CXCL12, CXCL9, CXCL13, CCL21, CCL27, CCL19, APP, C3, GAL, C3AR1). We believed these 10 genes are the main targets of HJD treatment of metastatic CM.

Correlation analysis showed that, except for CLL27, the other core gene are all related to the abundance of immune cells, demonstrating HJD treatment may modulate the abundance of immune cells in metastatic CM patients. Actually, the main compounds of HJD have also been found to have a regulatory role in regulating tumor immune mechanisms. Baicalin participates in the regulation of TLRs signaling pathways to exert immunomodulatory effects, thereby inhibiting the migration of cancer cells [36]. Berberine can inhibit the expression of PD-L1 in tumor cells and activate T cells to participate in tumor immune regulation [37, 38]. It was found that CXCL12 and CCL27 played an important role in CM metastasis, and were mainly involved in SKMC metastasis through the CXCR4-CXCR7-CXCL12 axis and CCL27-CCR10 axis [39, 40]. CXCL9 and CXCL13 have been confirmed to be overexpressed in both primary SKCM and metastatic CM [41, 42]. As ligands of CCR7, CCL19 and CCL21 play an important role in the migration of immune cells (T cells and dendritic cells), and deviation in the regulation of the CCR7-CCL19/CCL21 axis may lead to and cause the occurrence and metastasis of tumors [43]. C3, APP, GAL, and C3AR1 have also been shown to be involved in the regulation of cancer cell metastasis and tumor invasion in multiple cancers [44–47].

The prognostic analysis revealed that METASTATIC CM patients who had a poor prognosis with high levels of GAL, low levels of C3AR1, CXCL13, CXCL9, B cells, CD8+ cell, neutrophil, dendritic cells. Our study also revealed that the expression of core genes was associated with the abundance of certain immune cells and somatic copy number alterations of core genes could certainly regulate the immune cell infiltrations in metastatic CM. Limited studies were performed to explore the role of core genes in immune infiltration, so as to clarify the potential role of HJD in immune infiltration. Kim E's study found that Coptis can upregulate IFN-*γ* and other Th1 cell-related cytokines expression through the MAPK signaling pathway [48]. The elevated Th1 cytokine levels create an immuno-protective environment which thereby facilitates in curing cancer metastasis [49]. Therefore, our results add new insights into HJD's regulation of immune cell infiltration.

The enrichment analysis results show that HJD intervention and metastatic CM treatment involve multiple cancer signal pathways and signal pathways related to tumor metastasis, including melanoma, bladder cancer, prostate cancer, small cell lung cancer, chemokine signaling pathway, NF-kappa B signaling pathway, p53 signaling pathway, HIF-1 signaling pathway, PD-L1 expression and PD-1 checkpoint pathway in cancer. The HIF1 signaling pathway is currently believed to play a role in promoting the metastasis of multiple tumors [50]. Metastatic CM through its transcriptional regulator hypoxia-inducible factor 1 (HIF-1), vascular endothelial growth factor (VEGF), which promotes angiogenesis and metastasis of human cancerous cells [51]. The p53 and p53 signaling pathway are one of the center members of inhibiting the growth and metastasis of tumors [52], But the P53 signaling pathway is destroyed in metastatic CM [53]. At present, a large number of studies have proved that the activation of the NF-kappa B signaling pathway is one of the classic signal transmissions in cancer metastasis and inflammation [54, 55]. Cheng et al. studies have proved that the activation of the NF-kappa B signaling pathway plays an important role in CM metastasis and growth [56]. On the contrary, the inhibition of the NF-kappa B signaling pathway can effectively control SKCM metastasis [57].

We analyzed the potential mechanism of HJD to prevent CM metastasis based on immune infiltration, and found that HJD treatment of metastatic CM involves multiple cancer signaling pathways and cancer metastasis-related signaling pathways through enrichment analysis. Through prognostic analysis, we can think that HJD has the ability to improve the prognosis of metastatic CM patients and prevent cancer metastasis in CM patients. In summary, our results clarify the clinical significance and prognostic value of HJD in metastatic CM, reveal the potential mechanism of HJD to prevent the occurrence of metastasis in SKCM, and provide new methods and theories for the prognosis and treatment of CM and metastatic CM.

## 5. Conclusion

This research combines network pharmacology analysis and immune infiltration analysis, to explore and discover the potential mechanism of HJD to treat CM and prevent the metastasis of CM. It is found that HJD may effectively improve the bad prognosis of metastatic CM patients through prognostic analysis. Our research lays the foundation for the next HJD research in metastatic CM.

## Figures and Tables

**Table 1:** Information for the four GEO datasets included in the current study.

Dataset	Sample size (metastatic/primary)	Platform
GSE7553	40/14	GPL570
GSE8401	52/31	GPL96
GSE15605	12/47	GPL570
GSE46517	73/31	GPL96

**Table 2:** Top 10 core genes in PPI network.

Rank	Name	Score
1	CXCL12	3651006
2	CXCL9	3631224
3	CCL21	3631200
4	C3	3631058
5	CXCL13	3630960
6	APP	3630087
7	CCL19	3629640
8	C3AR1	3628801
9	CCL27	3628800
9	GAL	3628800

## Data Availability

The basic data of this study comes from public databases Drug data comes from HERB database (https://herb.ac.cn/) and TCMSP database (https://tcmspw.com/tcmsp.php) The protein interaction network database comes from the STRING database (https://www.string-db.org/) Disease database comes from GEO database (https://www.ncbi.nlm.nih.gov/geo/).
